# A New Species of Solitary *Meteorus* (Hymenoptera: Braconidae) Reared from Caterpillars of Toxic Butterflies (Lepidoptera: Nymphalidae) in Ecuador

**DOI:** 10.1673/031.009.3401

**Published:** 2009-06-02

**Authors:** Scott R. Shaw, Guinevere Z. Jones

**Affiliations:** Insect Museum, Department of Renewable Resources, University of Wyoming, 1000 East University Avenue, Laramie, Wyoming 82071-3354, U.S.A

**Keywords:** Neotropical, parasitoid, Ithomiinae, Solanaceae, taxonomy, behavior

## Abstract

A new species of parasitoid wasp, *Meteorus rugonasus* Shaw and Jones (Hymenoptera: Braconidae), is described from the Yanayacu Biological Station, Napo Province, Ecuador. The new species is diagnosed and compared to other species in the genus. It was reared from larvae of *Pteronymia zerlina* (Hewitson, 1855) (Lepidoptera: Nymphalidae, Ithomiinae) found feeding on leaves of Solanum (Solanaceae). The parasitoid is solitary. This is the first record of a *Meteorus* species attacking ithomiine Nymphalidae. A new species of parasitoid wasp, *Meteorus rugonasus* Shaw and Jones (Hymenoptera: Braconidae), is described from the Yanayacu Biological Station, Napo Province, Ecuador. The new species is diagnosed and compared to other species in the genus. It was reared from larvae of *Pteronymia zerlina* (Hewitson, 1855) (Lepidoptera: Nymphalidae, Ithomiinae) found feeding on leaves of *Solanum* (Solanaceae). The parasitoid is solitary. This is the first record of a *Meteorus* species attacking ithomiine Nymphalidae.

## Resumen

Una nueva especie, *Meteoros rugonasus* Shaw and Jones (Hymenoptera: Braconidae) es descrita para Yanayacu Estacion Biologia de Napo Provincia, Ecuador. La especie es analizada y comparada con otras especies del género. Los especimenes fueron criados de larvas de *Pteronymia zerlina* (Hewitson, 1855) (Lepidoptera: Nymphalidae, Ithomiinae), encontradas en hojas de *Solanum* (Solanaceae). El parasitoide es solitario.

## Introduction

**Figures 1–4.**
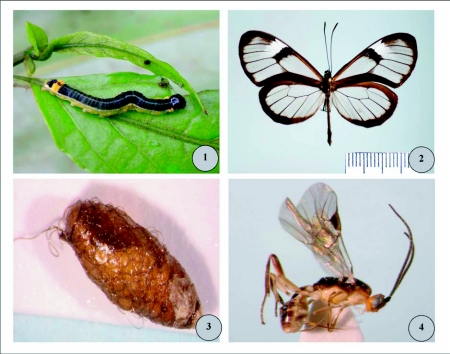
1) Host caterpillar of *Meteorus rugonasus: Pteronymia zerlina* (Hewitson, 1855) (Lepidoptera: Nymphalidae, Ithomiinae) on leaves of *Solanum* species (Solanaceae). 2) Adult male of *Pteronymia zerlina* (Hewitson, 1855) (Lepidoptera: Nymphalidae: Ithomiinae); Ecuador, Bolivar Prov., Balzabamba, 630 m, June 1938, leg. F. M. Brown (American Museum of Natural History). 3) Cocoon of *Meteorus rugonasus*, new species. 4) *Meteorus rugonasus*, new species, paratype female (lateral habitus).

*Meteorus* Haliday is a diverse and widespread genus of Braconidae comprising more than 250 described species worldwide (Muesebeck 1923, 1939; Nixon 1943; Huddleston 1980, 1983; Maetô 1990; Chen & Wu 2000; Zitani & Shaw 2002; Zitani 2003; Shaw & Nishida 2005; Stigenberg 2008). All are koinobiont endoparasitoids. Most attack young exposed-feeding Lepidoptera caterpillars, though some parasitize beetle larvae (Shaw & Huddleston 1991; Shaw 1995, 1997). *Meteorus* species are noted for their diverse silk-spinning and cocoon-forming behaviors (Zitani & Shaw 2002; Zitani 2003). While most are solitary parasitoids of small caterpillars, such as Geometridae, Noctuidae, and Pyralidae, several tropical *Meteorus* species are known to be gregarious parasitoids of larger caterpillars, including Sphingidae (Muesebeck 1939, 1958; Nixon 1943; Zitani et al. 1997, 1998; Zitani 2003; Shaw & Nishida 2005). Costa Rican *Meteorus* species have been the focus of recent taxonomic studies (Zitani et al. 1997, 1998; Shaw & Nishida 2005) but many Neotropical species remain undescribed. The purpose to this paper is to describe a new *Meteorus* recently found parasitizing caterpillars of *Pteronymia zerlina* (Hewitson, 1855) (Lepidoptera: Nymphalidae, Ithomiinae) on leaves of *Solanum* (Solanaceae) (Figure 1). This is the first record of *Meteorus* parasitizing ithomiine Nymphalidae. It is also the first record of a hymenopteran parasitoid utilizing *P. Zerlina* (Figure 2).

## Methods

### Taxonomy

Morphological terminology and characters used in this description follow Zitani et al. (1997, 1998) and Shaw & Nishida (2005). The holotype of *M. rugonasus* is deposited in the University of Wyoming Insect Museum (UWIM). Paratypes and associated cocoons are deposited at the UWIM, United States National Museum, Washington B.C. (USNM), the Museum of Comparative Zoology, Harvard University (MCZ), and the Museo Ecuatoriana de Ciencias Naturales (MECN).

### Biology

**Figures 5–10.**
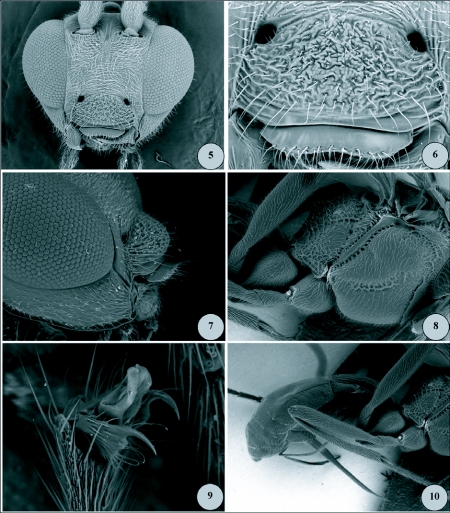
5) Scanning electron micrograph of *Meteorus rugonasus*, new species: head, anterior view, 180x. 6) Scanning electron micrograph of *Meteorus rugonasus*, new species: clypeus, anterior view, 600x. 7) Scanning electron micrograph of *Meteorus rugonasus*, new species: head, lateral view, 300x, showing protuberant and rugose clypeus. 8) Scanning electron micrograph of *Meteorus rugonasus*, new species: mesosoma, lateral view, 120x, showing mesopleuron and sternaulus sculpture. 9) Scanning electron micrograph of *Meteorus rugonasus*, new species: hind tarsal claws, 1000x. 10) Scanning electron micrograph of *Meteorus rugonasus*, new species: lateral habitus of mesosoma and metasoma, 80x, showing ovipositor.

The specimens for this study were reared from host caterpillars as part of the *Caterpillars and Parasitoids of the Eastern Andes of Ecuador* project (NSF-BSI-03-46729; NSF-BSI-07-17458; Dyer et al. 2007). Caterpillars of *Pteronymia zerlina* were collected during the months of August and September, 2005, and June, 2006, in the Yanayacu Biological Station and Center for Creative Studies (elevation 2163 m; 00°35.9′ S, 77°53.4′ W), which is situated on the northeastern slope of the Andes in Napo Province, Ecuador. The reserve comprises both primary and secondary growth montane forests (Greeney 2007). The Holdridge life zone is tropical montane moist forest (Holdridge 1967). Caterpillars were sampled by walking through various habitats and inspecting plants up to a height of 2.5 m. Second and third instar larvae of *Pteronymia zerlina* were collected while feeding on *Solanum* species (Solanaceae) (Figure 1). Upon discovery, caterpillars were collected in clear plastic bags with their food plant and transported to the rearing station. Caterpillars were assigned an identification code and the food plant was recorded. Rearing took place in plastic bags in an open air, covered shed, where caterpillars were exposed to ambient temperatures and day length, and fed their natural food plants. Frass and decaying plant material was removed every other day, and new plant material was provided as needed. Parasitoid cocoons were inspected daily and upon emergence the date was noted and adults were collected. *Meteorus* adults and cocoons were preserved in 75% ethanol. Digital images of host caterpillars were taken with a Nikon Coolpix camera. Scanning electron microscopy (Figures 5–10) was done at the University of Wyoming Microscopy Core Facility using a Hitachi tabletop scanning electron microscope, model TM-1000. Specimens were examined uncoated at an operating voltage of 15 kV. The host butterfly was identified by Keith Willmott based on adult specimens reared from the same caterpillar series.

### *Meteorus rugonasus* Shaw & Jones, new species (Figures 4–12)

***Diagnosis*:** head bright orange (Figure 4); mandible strongly twisted, teeth in lateral view with second directly behind first; clypeus convex, coarsely rugose, bulging (lateral view); ocelli small, ocello-ocular distance = 1.6x ocellar diameter; occipital carina complete; wing membrane clear; vein r 0.7x length of 3RSa; propodeum areolate-rugose; hind coxa smooth to finely granular; first metasomal tergite without dorsopes; ventral borders of tergum 1 joined completely along basal ½ of segment, fused medially without visible trace of suture; tergum 2 black laterally, basomedially with white elongate oval figure extending barely onto apex of tergum 1.

### Description of holotype female

**Figures 11–12.**
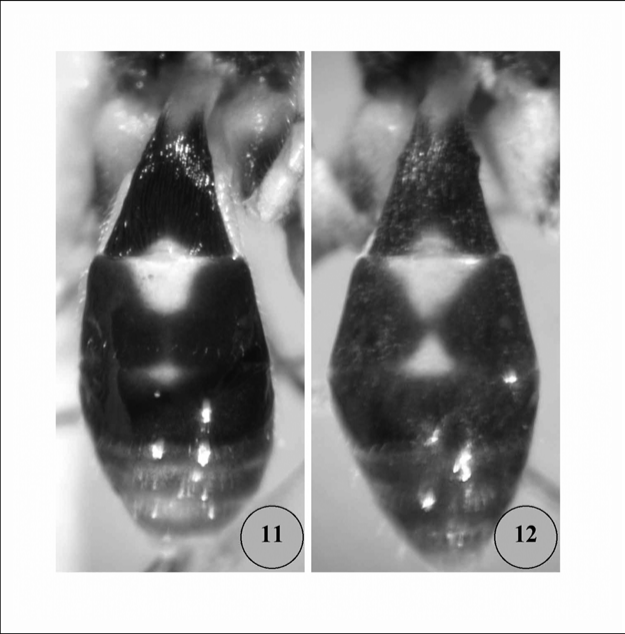
11) Color patterns of *Meteorus rugonasus*, new species: metasoma, dorsal view, holotype female. 12) Color patterns of *Meteorus rugonasus*, new species: metasoma, dorsal view, male.

***Body color*:** (Figures 4, 11). With contrasting light and dark markings as follows: head mostly bright orange, compound eye silver, antenna entirely dark brown; ocellar triangle black; dorsal margin of pronotum brownish orange, pronotum ventrally pale yellow; prosternum pale yellowish white; lateral lobes of mesonotum and lateral lobes of scutellum black, median lobe of mesonotum and scutellum orange; mesopleuron mostly pale yellow except dark brown dorsally below wings; metanotum and propodeum black; prothoracic and mesothoracic legs with similar color patterns: coxa and trochanter pale yellowish white, femur and tibia light brownish yellow with increasing brown pigment apically, tarsus light brown; metathoracic leg as follows: coxa with basal ½ yellowish white, apical ½ dark brown to black; trochanter and trochantellus pale yellowish white; femur in dorsal and external lateral view mostly dark brown, except yellowish brown at extreme base; femur in ventral or interior lateral view mostly pale yellow to pale brownish yellow; tibia and tarsus dark brown; wing membrane clear; wing venation dark brown; pterostigma entirely dark brown; metasomal tergites 1–3 black dorsally except petiole white basally in dorsal view, petiole entirely white ventrally, and tergum 2 medially with white elongate-oval marking extending onto apex of tergum 1 (Figure 11); metasoma ventrally mostly pale brownish yellow, apically yellowish white; ovipositor and sheaths dark brown.
***Body length*:** 4.5 mm.
***Head*:** (Figures 5–7) antenna comprising 28 flagellomeres; flagellar length/width ratios as follows: F1 = 3.7; F2 = 3.5; F3 = 3.3; F24 = 2.0; F25 = 2.0; F26 = 1.6; F27 = 1.5; F28 (apical flagellomere) = 2.5; tip of apical flagellomere acutely pointed; head 1.2x wider than high, head height 1.4x eye height; eye small but protuberant, slightly converging ventrally in anterior view (Figure 5); maximum face width 1.1 × minimum face width; minimum face width 1.1 × clypeus width; malar space length 1.3x mandible width basally; ocelli small, ocello-ocular distance 1.6x ocellar diameter; clypeus convex, coarsely rugose (Figure 6), bulging in lateral view (Figure 7); mandible strongly twisted; occipital carina complete; vertex, in dorsal view, descending vertically behind lateral ocelli.
***Mesosoma*:** (Figure 8) notauli rugulose, not distinct, and mesonotal lobes not well-defined; scutellar furrow with 1 median carina; mesopleuron polished, punctate; sternaulus rugulose, broad but not long (Figure 8); propodeum areolate-rugose, median depression absent.
**Legs:** Metathoracic coxa smooth to finely granular; larger hind tibial spur about ½ as long as hind basitarsus; tarsal claw with a small blunt basal tooth, strongly curved (Figure 9).
***Wings*:** forewing length 4.5mm; vein m-cu postfurcal; second submarginal cell of forewing not strongly narrowed anteriorly; vein r 0.7x length of 3RSa.
***Metasoma*:** (Figure 10) first metasomal tergite without dorsopes; ventral borders of tergum 1 joined completely along basal ½ of segment; tergum 1 dorso-longitudinally costate in apical half beyond spiracles, costae slightly convergent posteriorly; ovipositor long, thick at base, 2.1x longer than tergum 1.
***Variation*:** other females as in holotype except body length = 4.0–5.0mm; forewing length = 4.2–4.7mm; antennae with 28–29 flagellomeres. Body color same as holotype except metasomal tergites 1–3 medium brown to black; white elongate oval marking on tergite 2 sometimes smaller or nearly absent; median lobe of mesonotum and scutellum sometimes dark orange-brown (yellow in holotype); scutellar sulcus sometimes dark brown (yellow in holotype).
***Males*:** similar to females except smaller, body length = 3.7–4.6mm. Antenna with 27–28 flagellomeres. Body color similar to holotype except head dull brownish orange; prosternum darker yellow or light brown; with a white “hourglass” marking on tergum 2 (Figure 12); median lobe of mesonotum and scutellum medium brown; prothoracic and mesothoracic legs with similar color patterns: coxa and trochanter light brown, femur and tibia medium brown with increasing brown pigment apically.
***Holotype*:** female (point-mounted), Ecuador: Napo Province, Yanayacu Biological Station, S 00°35.9′, W 77°53.4′, reared at 2163 m, [caterpillar voucher No.] YY-6945, 04 September 2005, leg. R. B. Granizo & M. R. Simbaña, Plot 91, Yanyacu forest, collected at 2122 m, host plant: Solanaceae, *Solanum* species, ex. Nymphalidae, instar 3, parasitoid pupated (?) September 2005, adult wasp emerged 05 October 2005, solitary parasitoid. Deposited in UWIM.
***Paratypes*:** 3 ♀♀, same data as holotype except YY-6947, YY-6948, YY-6949, pupated 20 September 2005, adult wasps emerged 04, 05 and 06 October 2005. 3 ♀♀♀, 6 ♂♂, same data as holotype except YY-6855, YY-6856, YY-6857, YY-6858, YY-6859, YY-6860, YY-6862, YY-6863, YY-6864, collected 31 August 2005, Plot 87, Camino Pumayacu, collected at 2110 m, host instar 2, adult wasps emerged 13, 14, and 24 October 2005. 1 ♀, same data except YY-14996, Camino Pumayacu, host instar 3, parasitoid pupated 17 June 2006, adult wasp emerged 07 July 2007. (7 ♀♀, 6 ♂♂ total) Deposited in UWIM, USNM, MCZ, MECN.
***Cocoon*:** (Figure 3) elongate-oval, tapering at anterior end to rounded point, dark brown with loosely spun exterior silk (resembling coarse baling wire) appearing whitish yellow. Exit cap cut neatly away in a complete circle, completely detaching cap from cocoon body. Cocoon with short suspending thread of indeterminate length (in each case the suspending thread was either tangled or broken). In two cases the cocoon does not appear to have any suspending thread, and no broken thread is apparent.

### Comments

*Meteorus rugonasus* NEW SPECIES is most similar to *Meteorus uno* Zitani and *Meteorus oviedoi* Shaw & Nishida. All three species exhibit a bright orange head, extensive black body markings, a rugose clypeus, and a metasomal tergum 1 that is fused ventrally into a smooth tubular petiole (basally), this lacking any trace of a ventro-medial suture. These characters are unique states that could potentially be interpreted as synapomorphies uniting these three species into a monophyletic group. In the case of *M. rugonasus*, the clypeus is more coarsely rugose and more distinctly convex than in the other two species. In the key to Costa Rican *Meteorus* (Zitani et al. 1998) *M. rugonasus* keys to couplet 8 (near *M. uno*) but it can easily be distinguished from *M. uno* by the complete occipital carina (*Meteorus oviedoi*, described since 2005, also keys to couplet 8 in Zitani's key). Obvious color differences distinguish these species, but *M. rugonasus* most closely resembles *M. oviedoi*. The mesonotum of *M. uno* is uniformly orange, while the mesonota of *M. oviedoi* and *M. rugonasus* have black lateral lobes. The hind coxa is entirely black in *M. uno* but black apically in *M. oviedoi* and *M. rugonasus*. The dorsal surface of the propodeum is black in *M. uno*, while the propodeum is entirely black in *M. oviedoi* and *M. rugonasus*. Metasomal tergum 2 is entirely black in *M. uno*, while in *M. oviedoi* tergum 2 is white medially and black laterally, forming a broad hourglass-shaped figure medially. *Meteorus rugonasus* differs from both by having the medial surface of tergum 2 with a white elongate-oval marking, this extending onto apex of tergum 1. *Meteorus rugonasus* is a solitary parasitoid of ithomiine Nymphalidae, while *M. oviedoi* is a gregarious parsitoid of Limacodidae (Shaw & Nishida 2005). The host of *M. uno* is unknown (Zitani et al. 1998).

The discovery of a *Meteorus* attacking Ithomiinae is of interest because this butterfly subfamily was not a previously known host. Other Nymphalidae have been recorded as hosts for two European *Meteorus* species (Huddleston 1980; Stigenberg 2008).

***Etymology*:** This species name, from the Latin *rugosus* meaning “wrinkled” and Latin *nasus* meaning “nose,” refers to the prominent, coarsely wrinkled clypeus of *M. rugonasus* (Figures 5–6), which in lateral profile appears like a wrinkled nose (Figure 7). It is also a play on words, insofar as one might “wrinkle one's nose” in response to a nasty odor; the host caterpillar is presumed to possess toxic alkaloids.

### Distribution

The type series of *M. rugonasus* was reared from *P. zerlina* larvae collected in the ecological reserve of the Yanyacu Biological Station and Center for Creative Studies, which is situated on the northeastern slope of the Andes in Napo Province, Ecuador. This is the only location where the wasp species has been found, although its host butterfly is more widely distributed in Ecuador, Peru, Colombia, and Venezuela (Racheli & Racheli 2001; Willmott & Mallett 2004).

### Biology and rearing records

The hosts are second and third instar caterpillars of *Pteronymia zerlina* feeding on *Solanum* species (Solanaceae). Ithomiinae (a strictly Neotropical butterfly lineage) is a new host subfamily record for Meteorinae, a diverse braconid subfamily with at least 278 described species worldwide (Zitani 2003). The caterpillar of *P. zerlina is* smooth and boldly colored, with a black head capsule, a broad black dorsum bearing narrow white median stripe, pale greenish-yellow lateral regions, and a bright yellow-orange dorsal spot on A7 (Figure 1). The caterpillars are solitary, feeding and resting conspicuously exposed on *Solanum* leaves. The presumed toxicity of the host caterpillar is supported by its aposematic coloration, the known toxicity of its solanaceous host plant, and the known toxicity of other ithomiine larvae (DeVries 1987; Trigo 2000).

*M. rugonasus* is a solitary koinobiont endoparasitoid. The number of individuals that emerged per host was consistently one (mean = 1, n = 14). The observed ratio of females to males emerging was 8:6.

## Discussion

Many Neotropical Lepidoptera are known to be unpalatable to vertebrate predators, but our understanding of the underlying chemical mechanism is often poor (Trigo 2000). The distasteful properties of the ithomiines have long been presumed to be due to larval feeding on the plant family Solanaceae, known as the deadly nightshades (DeVries 1987). The Solanaceae contain a variety of toxic compounds used historically as sources of medicines and poisons. More recently it has been discovered that defensive pyrrolizidine alkaloids found in the adult butterflies are acquired during adult flower-feeding and not during larval development (Brown 1984).

Ithomiine butterflies (Figure 2) are also well known participants in Müllerian mimicry rings (DeVries 1987; Willmott & Freitas 2006). Müllerian mimicry theory suggests that aposematic species in a local community should converge towards a common defensive warning signal. The reality is more curious: ithomiines in one community may display eight or more warning patterns, each belonging to a different mimicry ring (Willmott & Mallett 2004). Several explanations for polymorphism in wing patterns have been suggested including developmental constraints, weak selective pressure for pattern convergence, rapid evolution of new patterns, and the idea that host-plant microhabitat is constraining habitat partitioning by adults (Willmott & Mallett 2004). Our discovery of parasitoids of larval ithomiines raises an alternative possibility: differential mortality rates caused by larval parasitoids might be affecting the survivorship of particular wing pattern phenotypes in ways that are independent from adult mortality.

The discovery of a *Meteorus* species utilizing ithomiine larva raises several interesting questions. Presumably, the larvae of *Pteronymia zerlina* are sequestering defensive chemicals from their solanaceous food plants *(Solanum)*. If *Pteronymia* caterpillars contain toxic chemicals, then how are *Meteorus* larvae able to utilize them as food? Are the *Meteorus* larvae, like their hosts, somehow metabolizing secondary chemicals? The fact that *M. rugonasus* has a bright orange head might be interpreted as aposomatic coloration (Figure 4). It seems possible that *M. rugonasus* is chemically-defended. It could also be possible that the *Meteorus* larvae are behaviorally avoiding defensive chemicals. For example, the level of defensive chemicals varies among individual *Pteronymia* larvae, *Meteorus* females might be able to assess this while searching for hosts, and they might be preferentially selecting caterpillars with lower levels of chemical defenses. If the chemical defenses of the caterpillar are situated mainly in the blood, it may be possible that the *M. rugonasus* may be avoiding contact with the toxins by inserting the egg into muscles or other organ tissue. The growing wasp larva may be avoiding contact with toxins by feeding preferentially inside muscles or other organ tissue. It would be interesting to examine the chemical ecology of both the host caterpillar and the parasitoid in an attempt to resolve these issues, and to test whether the particular host plant indeed contains toxins.

## Editor's note

Paper copies of this article will be deposited in the following libraries. Senckenberg Library, Frankfurt Germany; National Museum of Natural History, Paris, France; Field Museum of Natural History, Chicago, Illinois USA; the University of Wisconsin, Madison, USA; the University of Arizona, Tucson, Arizona USA; Smithsonian Institution Libraries, Washington D.C. USA; The Linnean Society, London, England.
